# RustMAE: A Spatiotemporal Transformer for Short- to Medium-Term Warning of Wheat Stripe Rust Spring Spread

**DOI:** 10.1016/j.isci.2026.115666

**Published:** 2026-04-09

**Authors:** Xinde Zhang, Xiaoyu Wan, Yuneng Du, Shaowen Li

**Affiliations:** 1 Institute of Industrial Crops, Anhui Academy of Agricultural Sciences (AAAS), Hefei, Anhui 230001, China; 2 School of Mathematics and Statistics, Hefei Normal University, Hefei, Anhui 230601, China; 3 School of Economics and Management, Anhui Agricultural University, Hefei, Anhui 230036, China; 4 School of Information and Artificial Intelligence, Anhui Agricultural University, Hefei 230036, China

**Keywords:** plant pathology, agricultural science, agricultural engineering, agricultural instrumentation, artificial intelligence, artificial intelligence applications

## Abstract

Wheat stripe rust poses a significant threat to food security in China. This study proposes RustMAE, a spatiotemporal Transformer-based early warning method utilizing masked autoencoders for 14-day disease spread forecasting during the spring epidemic season. RustMAE employs a dual-branch MAE pre-training framework that integrates spectral reconstruction and spatial structure recovery to learn disease-relevant features from large-scale unlabeled remote sensing imagery. A multi-scale spatiotemporal attention mechanism captures disease evolution patterns across 3-, 7-, and 14-day windows. By fusing optical imagery, synthetic aperture radar, and meteorological factors, the model constructs a multi-source driven disease risk prediction system. Validation on 1,847 samples from Shaanxi, Gansu, and Henan provinces (2019–2024) demonstrates that RustMAE achieves 87.23% overall accuracy and 0.924 AUC, significantly outperforming benchmark methods. This approach provides an innovative pathway for precise wheat stripe rust early warning and control.

## Highlights

• Self-supervised MAE learns disease features from 120k unlabeled images
• Multi-scale Transformer captures disease evolution via 3/7/14-day windows
• Delivers 14-day warning with 87% OA, enabling timely interventions
• Fuses optical/SAR/meteorological data for robust predictions under clouds

## Graphical abstract

**Figure:**
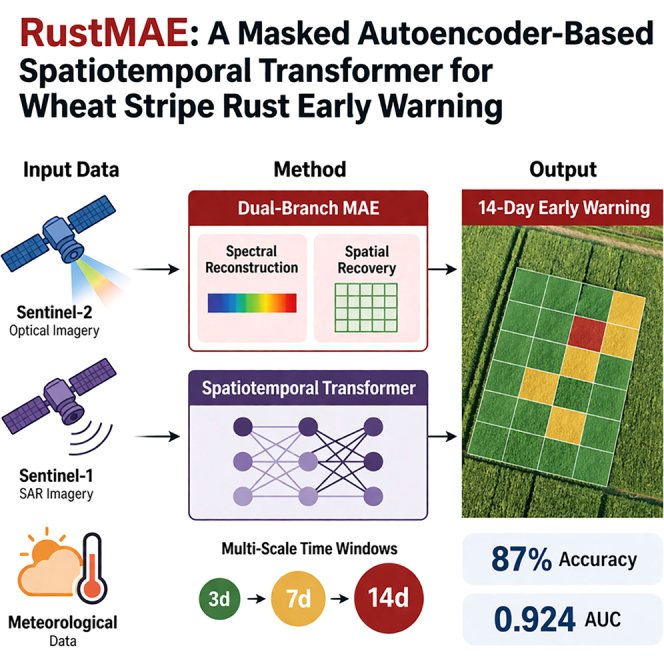

## Introduction

Wheat (*Triticum aestivum* L.) is among the world’s most widely cultivated food crops, serving as a dietary staple for billions of people. The stability of wheat production is therefore inextricably linked to global food security and social stability. However, wheat production faces severe threats from stripe rust, caused by the fungus *Puccinia striiformis f. sp. tritici*. This airborne pathogen is characterized by rapid spread, extensive epidemic range, and strong outbreak potential; in severe epidemic years, yield losses can exceed 40%, resulting in complete crop failure in affected regions. China represents one of the most severely impacted nations, with provinces including Shaanxi, Gansu, Sichuan, and Henan experiencing large-scale spring epidemics annually, affecting over 4 million hectares and causing billions of yuan in economic losses.

Traditional stripe rust monitoring has relied predominantly on labor-intensive field surveys and visual interpretation by plant protection personnel. These approaches are costly, time-consuming, and inherently limited in spatial coverage and temporal frequency, often detecting outbreaks only after disease proliferation has occurred, thereby missing the optimal window for chemical intervention. In recent years, remote sensing technology has emerged as a transformative alternative for regional-scale crop health monitoring, offering wide coverage, short revisit periods, and relatively low acquisition costs. These advantages have catalyzed extensive applications in precision agriculture and plant phenotyping, providing unprecedented capabilities for non-destructive, large-scale disease observation.

Recent advances in deep learning have catalyzed extensive research on remote sensing-based stripe rust detection. Tang et al. developed the RustNet classifier based on ResNet-18 architecture, achieving AUC values of 0.72–0.87 for field RGB image classification.^1^ Deng et al. employed convolutional neural networks with high-resolution UAV imagery to achieve pixel-level segmentation of transmission centers under complex field conditions, with F1 scores reaching 0.81.^2^ Zhao et al. integrated Sentinel-2 multispectral data with meteorological elements using geographical detectors for feature selection, constructing a regional-scale monitoring model based on random forest (RF).^3^ Pan et al. applied the PSPNet semantic segmentation network to UAV images, achieving 98% overall classification accuracy.^4^ Liu et al.^5^ developed the StripeRust-Pocket mobile application integrating StripeRustNet for real-time severity assessment on smartphones, with lesion segmentation mIoU reaching 86.08%5. Schirrmann et al.utilized deep residual networks for early detection in close-range canopy images.^6^

Concurrently, the rise of self-supervised learning paradigms has brought new breakthroughs to remote sensing image feature extraction, offering solutions to the bottleneck of limited annotated data in agricultural applications. Lin et al. proposed SS-MAE, a spatial-spectral masked autoencoder (MAE) that significantly improved multi-source remote sensing image classification performance through self-supervised pre-training strategies.^7^ Reed et al. designed the Scale-MAE method targeting the multi-scale characteristics of remote sensing images, achieving accuracy improvements of 2.4%–5.6% in land cover segmentation tasks.^8^

While the above research has laid a solid foundation for stripe rust remote sensing monitoring, existing methods predominantly focus on detection and recognition after disease occurrence, lacking early warning capabilities before disease outbreak. Deng et al. proposed the RustQNet multi-modal deep learning framework for quantitative inversion of the stripe rust disease index, achieving precise severity assessment.^9^ Comprehensive reviews by Zhu et al. and Jafar et al. have systematically documented advances in UAV-based and AI-driven plant disease detection, respectively.^10^^,^^11^ However, research on deep modeling of stripe rust spatiotemporal evolution patterns remains relatively weak, and the disease development trend information contained in multi-temporal remote sensing sequences has not been fully exploited.

To address these limitations, self-supervised learning methodologies have been increasingly adopted in agricultural remote sensing. Wang et al. introduced feature-guided MAE (FG-MAE), utilizing histogram of oriented gradients and normalized difference vegetation index as prior information to guide mask strategy generation, achieving significant performance improvements in multispectral and SAR image classification tasks.^12^ Fan et al. designed the MAPM method integrating position prediction for polarimetric SAR image classification, adding position prediction auxiliary tasks on top of mask reconstruction to enhance spatial distribution perception.^13^ Guo et al. constructed SkySense, a multi-modal remote sensing foundation model pre-trained on over 21.5 million images covering optical, SAR, and infrared data types.^14^ Empirical studies by Santos et al. demonstrated that self-supervised pre-trained models offer particular advantages in few-shot learning scenarios for crop monitoring, while Safonova et al. further showed that using only 5% of annotated data, self-supervised methods can achieve or exceed the classification accuracy of fully supervised learning.^15^^,^^16^

Parallel advances in spatiotemporal modeling have positioned Transformer architectures as powerful tools for capturing disease dynamics. The vision transformer (ViT) and its variants have been widely adopted in agricultural applications. Bi et al. applied Transformers to soybean yield prediction, constructing spatiotemporal feature representations based on time-series remote sensing images, with RMSE reduced by 40% compared to traditional machine learning methods.^17^ Li et al. proposed the lightweight PMVT network, adapting the MobileViT architecture to mobile devices for real-time plant disease recognition and classification.^18^ Barman et al. developed the ViT-SmartAgri system for disease detection based on smartphone-captured field images, achieving 90.99% overall recognition accuracy.^19^ Parez et al. reviewed efficient ViT applications in plant disease detection from a visual intelligence perspective, analyzing model lightweighting and attention mechanism optimization.^20^ Wen et al. provided a comprehensive survey on Transformer time series modeling at IJCAI, reviewing temporal attention, frequency domain decomposition, and sparse attention technologies.^21^ Fu et al. proposed the STF-MoE model for wheat yield estimation, integrating LSTM’s temporal modeling with Transformer’s global attention mechanism via dynamic gating strategies.^22^ Xu et al. systematically reviewed Transformer-based long-term forecasting methods, highlighting position encoding design and multivariate dependency modeling as core research directions.^23^

For disease prediction specifically, integrating spatiotemporal modeling with epidemiological understanding is essential for constructing effective early warning systems. Delfani et al. analyzed integrated applications of IoT, machine learning, and AI for disease forecasting under climate change, emphasizing that multi-source heterogeneous data fusion and spatiotemporal modeling improvements are keys to precise early warning.^24^ Lee and Yun validated deep learning methods for pest and disease risk prediction using sequential environmental data, demonstrating recurrent neural networks' effectiveness in capturing temporal evolution patterns of meteorological drivers.^25^ Ashfaq et al. constructed artificial neural network models linking meteorological factors to stripe rust and powdery mildew severity, demonstrating high prediction accuracy.^26^ Massah Bavani et al. conducted systematic reviews categorizing prediction approaches into statistical regression, machine learning, deep learning, and mechanistic models, noting that combining data-driven methods with domain knowledge improves reliability.^27^ Yu et al. explored application prospects of time series prediction technology in precision plant protection, identifying the integration of remote sensing observations with meteorological forecasts for multi-source spatiotemporal modeling as a major development direction.^28^

Concurrently, the field has witnessed rapid advances in remote sensing foundation models. SatMAE++ introduced temporal embeddings and multi-scale pre-training strategies for optical and multi-spectral imagery.^29^^,^^30^ Scale-MAE further addresses multi-scale geospatial representation learning by explicitly modeling relationships across different spatial resolutions.^31^ AgriFM proposed a multi-source temporal foundation model pre-trained on over 25 million image samples from MODIS, Landsat, and Sentinel-2 for crop mapping.^32^ The Prithvi series represents significant efforts in large-scale pre-training for Earth observation.^33^ For SAR imagery, SARMAE introduced noise-aware representation learning with speckle-aware enhancement and semantic anchor constraints.^34^ For satellite image time series (SITS), UTAE modified temporal attention mechanisms for efficient semantic segmentation, while SITSMamba leveraged the Mamba architecture with dual-branch decoders for crop classification, achieving efficient temporal modeling with linear complexity compared to Transformers' quadratic attention.^35^^,^^36^

Despite these substantial advances, critical research gaps persist. Most existing work focuses on post-occurrence detection, lacking the capability to forecast disease evolution during the latent-to-symptomatic transition period. Specifically, current approaches inadequately address: (1) utilization of self-supervised pre-training to learn robust, disease-relevant features from unlabeled remote sensing imagery; (2) explicit modeling of multi-scale temporal dynamics via spatiotemporal Transformers to capture disease evolution patterns across 3-, 7-, and 14-day windows; and (3) integration of optical, SAR, and meteorological data within a single architecture for practical 14-day advance warning.

**Figure 1:**
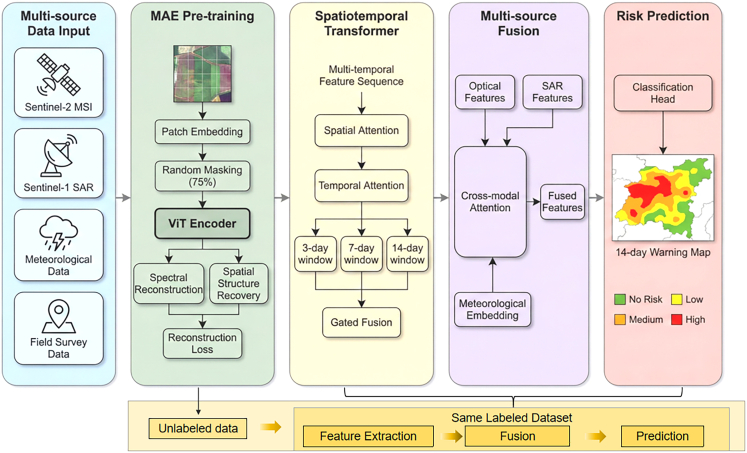
Overall research framework of the RustMAE wheat stripe rust early warning method The framework consists of two main phases: (1) Self-supervised pre-training phase (Module 2): A dual-branch masked autoencoder (MAE) is pre-trained on a large-scale unlabeled Sentinel-2 image dataset to learn general spectral-spatial feature representations. (2) Supervised fine-tuning and warning phase (Modules 3–5): The pre-trained encoder weights are transferred to initialize the spatiotemporal transformer. Multi-temporal Sentinel-2 and Sentinel-1 imagery from the labeled dataset are processed by the spatiotemporal transformer (Module 3) to extract dynamic features. These features are then fused with meteorological data via a cross-modal attention fusion module (Module 4). Finally, the fused multi-modal features are fed into the warning model (Module 5) to generate 14-day stripe rust risk predictions. The entire process is end-to-end from raw data input to risk map output.

**Figure 2:**
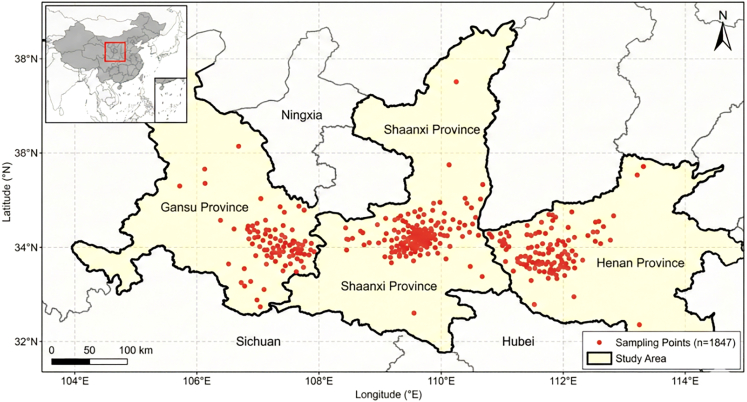
Schematic map of geographical location and sampling point distribution in the study area Map shows the geographical location of the study area (104°-114°E, 32°–38°N) and the spatial distribution of 1,847 ground survey points across Shaanxi, Gansu, and Henan provinces.

**Figure 3:**
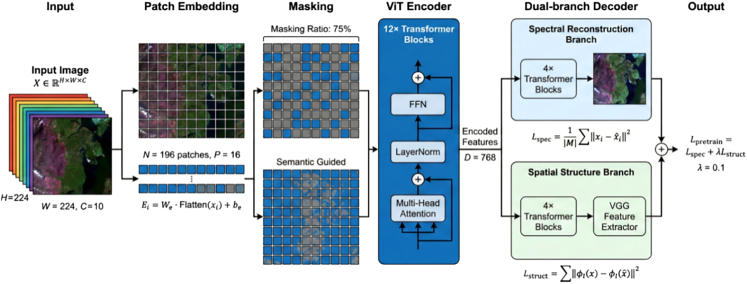
Structure diagram of dual-branch MAE pre-training framework Schematic diagram of the dual-branch MAE pre-training framework integrating spectral reconstruction and spatial structure recovery for learning feature representations from unlabeled remote sensing imagery.

**Figure 4:**
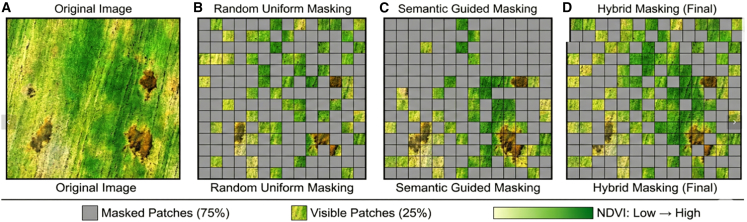
Visualization of the hybrid masking strategy Illustration of the hybrid masking strategy combining random uniform masking (75% ratio) and semantic-guided masking based on NDVI values to focus reconstruction on vegetation-dense regions during MAE pre-training.

To bridge these gaps, this study proposes RustMAE, a MAE-based spatiotemporal Transformer methodology for wheat stripe rust early warning. The model integrates optical imagery, synthetic aperture radar, and meteorological factors (Table 1), capturing disease evolution across 3-, 7-, and 14-day windows (Table 2). The overall research framework is illustrated in Figure 1, with validation conducted across the wheat-growing regions of Shaanxi, Gansu, and Henan provinces (2019--2024; Figure 2). The framework consisting of two primary phases: (1) a self-supervised pre-training phase employing a dual-branch MAE to learn general spectral-spatial representations from large-scale unlabeled Sentinel-2 imagery (Figure 3), and (2) a supervised fine-tuning and warning phase wherein pre-trained weights initialize a spatiotemporal Transformer that processes multi-temporal Sentinel-2 and Sentinel-1 sequences. The pre-training employs a hybrid masking strategy prioritizing vegetation-dense regions (Table 3), as illustrated in Figure 4. Validation on 1,847 temporally aligned survey points (Table 4) with high observational fidelity to target dates (Table 5) demonstrates that RustMAE achieves 87.23% overall accuracy and 0.924 AUC, significantly outperforming benchmark methods. The principal innovations include: (1) a dual-branch MAE pre-training framework integrating spectral reconstruction and spatial structure recovery to enhance the perception of early disease stress features; (2) a multi-scale spatiotemporal attention mechanism that adaptively aggregates disease evolution information across varying temporal and spatial scales; and (3) validation of 14-day advance risk forecasting capability through multi-source data fusion, significantly outperforming RF, ResNet-50, ViT-Base, and ViViT benchmarks (87.23% accuracy, 84.77% F1-score, 0.924 AUC). This methodology establishes a novel technical pathway for precise early warning and tactical intervention of wheat stripe rust, bridging the critical gap between disease detection and predictive prevention.

**Table 1:** Multi-source data parameter configuration

Data Type	Data Source	Spatial Resolution	Temporal Resolution	Bands/Elements	Time Range
Multispectral imagery	sentinel-2	10-20 m	5 days	B2-B12, 10 bands total	2019–2024
SAR imagery	sentinel-1	10 m	12 days	VV, VH polarization	2019–2024
Meteorological data	CIMISS	1 km	daily	temperature, humidity, precipitation, and so forth.	2019–2024
Disease survey	provincial plant protection stations	point location	weekly	affected area, severity	2019–2024

**Table 2:** Establishing the temporal correspondence between training samples and the early warning window

Warning Window	Input Data End Date	Input Data Period	Prediction Target	Temporal Offset
3-day	D - 3 days	D-18 to D-3 (15 days)	day D	3 days
7-day	D - 7 days	D-22 to D-7 (15 days)	day D	7 days
14-day	D - 14 days	D-29 to D-14 (15 days)	day D	14 days

**Table 3:** Quantitative specification of hybrid masking strategy

Masking Component	Patch Selection Criterion	Proportion of Patches	Masking Ratio Applied	Contribution to Overall 75% Masking
Semantic-guided (high vegetation)	NDVI >0.5 (top 60% by NDVI)	60%	85%	51% (0.6 × 0.85)
Random (low vegetation)	NDVI ≤0.5 (bottom 40% by NDVI)	40%	60%	24% (0.4 × 0.60)
Total	–	100%	75% (weighted average)	75%

**Table 4:** Statistical information of wheat stripe rust warning dataset

Statistical Indicator	Value
Total survey points	1847
Time span	2019–2024
Provinces covered	Shaanxi, Gansu, Henan
Total image scenes	11082
No risk samples	687 (37.2%)
Low risk samples	524 (28.4%)
Medium risk samples	412 (22.3%)
High risk samples	224 (12.1%)
Training set ratio	70%
Validation set ratio	15%
Test set ratio	15%

**Table 5:** The actual time distribution observed in the dataset

Metric	Value
Average temporal offset from the target date	1.2 days
Observations within ±1 day of the target	73.4%
Observations within ±2 days of the target	91.7%
Observations requiring interpolation	8.3%
SAR data used to fill optical gaps	12.4% of time steps

## Results

### MAE pre-training enhances feature discrimination

**Figure 5:**
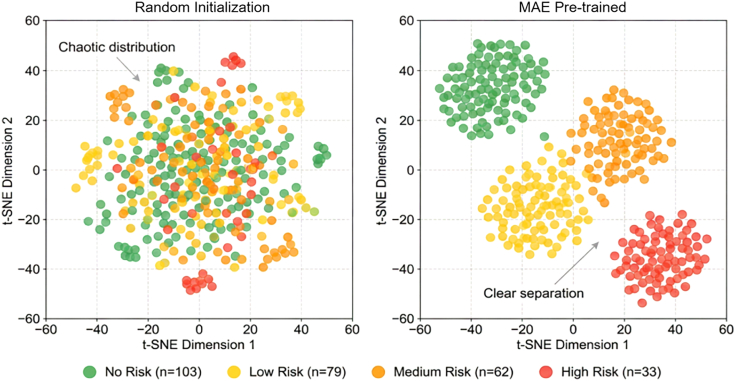
t-SNE visualization comparison of feature space before and after pre-training t-SNE visualization comparing feature distributions before (random initialization) and after dual-branch MAE pre-training, showing the transition from highly mixed, overlapping risk-level distributions to distinct clusters with clear separation between high-risk and no-risk samples.

To validate the effectiveness of MAE self-supervised pre-training for stripe rust feature learning, we conducted a comparative analysis of feature representation capabilities before and after pre-training. Using t-SNE dimensionality reduction, we projected high-dimensional encoder outputs into two-dimensional space for visualization (Figure 5).

Features extracted by the randomly initialized encoder without pre-training exhibited a highly mixed distribution, with samples of four risk levels overlapping extensively in feature space and blurred inter-class boundaries. In contrast, after dual-branch MAE pre-training, samples of the same risk level showed obvious clustering trends, with high-risk samples forming clear separation zones from no-risk samples. Although some overlap persisted between medium-risk and low-risk samples, the overall clustering structure improved significantly, indicating that MAE pre-training effectively learns discriminative feature representations related to stripe rust risk.

**Figure 6:**
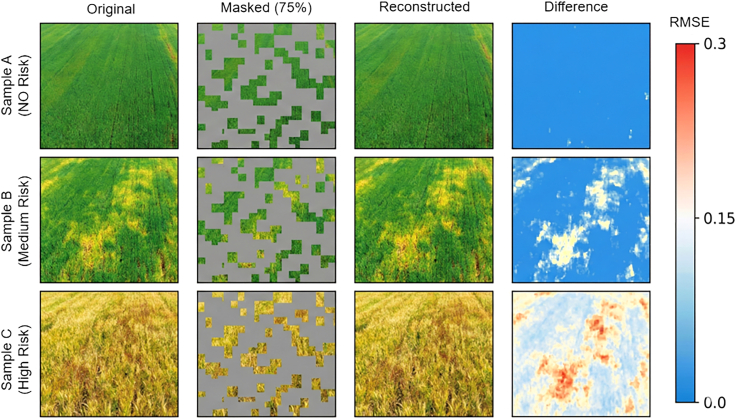
MAE reconstruction effect visualization (original-masked-reconstructed comparison) Conceptual illustration of the MAE reconstruction process shows original, masked (75%), and reconstructed images of wheat stripe rust; actual pre-training was performed on Sentinel-2 satellite imagery (10–20 m resolution), with this high-resolution field photograph used for visual demonstration purposes only.

The reconstruction loss convergence curves demonstrated that the spectral reconstruction branch rapidly decreased and stabilized within the first 50 epochs, while the spatial structure branch converged more slowly, reaching stability around 100 epochs. This dual-branch joint training enabled the model to simultaneously acquire capabilities in spectral detail recovery and spatial semantic understanding. Reconstruction visualizations (Figure 6) showed that despite 75% of image regions being randomly masked, the model accurately recovered spectral features and spatial textures of vegetation-covered areas. In stripe rust occurrence areas, reconstructed images preserved color change characteristics caused by the disease, indicating that the pre-trained encoder possesses perceptual capability for stripe rust-related spectral anomalies.

### Superior performance compared to baseline methods

We compared RustMAE against six representative baseline methods: RF and support vector machine (SVM) as traditional machine learning approaches; ResNet-50 as a deep convolutional neural network baseline; ViT-Base as a standard ViT; TimeSformer for video Transformer temporal modeling; and ViViT for spatiotemporal Transformer pre-training. All deep learning methods were initialized with ImageNet pre-trained weights and fine-tuned on our dataset.

RustMAE achieved optimal performance across all evaluation metrics (Table 6). Overall accuracy (OA) reached 87.23%, with a precision of 85.18%, a recall of 84.37%, an F1-score of 84.77%, and an AUC of 0.924. Compared to traditional machine learning methods, RustMAE improved OA by 15.89% over RF (71.34%) and 17.45% over SVM (69.78%), highlighting the advantage of deep learning models' nonlinear feature extraction capabilities.

**Table 6:** Performance comparison between RustMAE and baseline methods

Method	OA (%)	Precision (%)	Recall (%)	F1 (%)	AUC
RF	71.34	68.52	65.87	67.17	0.812
SVM	69.78	66.41	64.23	65.3	0.798
ResNet-50	76.89	74.36	72.18	73.25	0.856
ViT-Base	79.45	77.82	75.64	76.71	0.873
TimeSformer	82.67	80.35	78.92	79.63	0.894
ViViT	83.91	81.74	80.56	81.14	0.901
RustMAE	87.23	85.18	84.37	84.77	0.924

Compared to ResNet-50 (OA 76.89%), RustMAE improved OA by 10.34% and F1-score by 11.52%, demonstrating the Transformer architecture’s superiority over convolutional neural networks in capturing long-range dependencies in remote sensing imagery. Although ViT-Base adopted the same Transformer architecture, its lack of temporal modeling mechanisms resulted in lower performance (OA 79.45%) compared to spatiotemporal Transformer methods TimeSformer (82.67%) and ViViT (83.91%). RustMAE’s improvement over ViViT (OA +3.32%, F1 +3.63%) primarily benefited from the dual-branch MAE pre-training strategy and multi-scale spatiotemporal attention mechanism designed specifically for stripe rust monitoring tasks.

**Figure 7:**
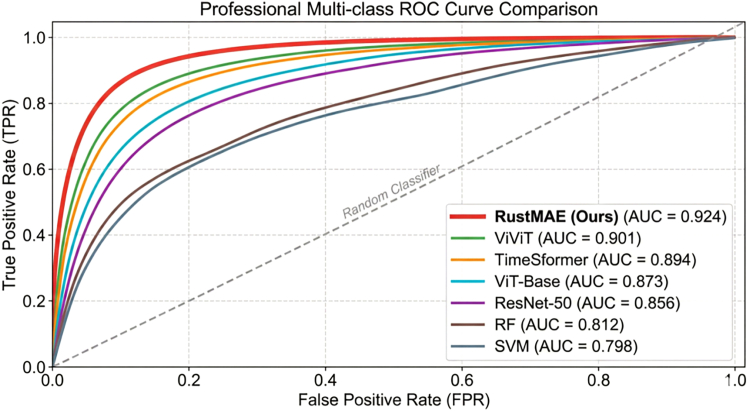
ROC curve comparison of each method Receiver operating characteristic (ROC) curves compare RustMAE (AUC = 0.924) with baseline methods, demonstrating superior classification performance with the curve positioned closest to the upper-left corner.

ROC curve analysis (Figure 7) showed RustMAE’s curve closest to the upper left corner, demonstrating optimal classification performance with the highest true positive rate at any given false positive rate.

### Detailed risk level classification performance

The analysis of classification performance across different risk levels (Table 7) revealed that the no-risk category achieved the highest F1-score (92.44%), attributable to sufficient sample size (*n* = 103) and relatively stable spectral features. Although the high-risk category contained the smallest sample size (*n* = 33), recall still reached 82.09%, indicating robust recognition capability for severely diseased areas—critical for practical early warning applications. Some confusion existed between low-risk and medium-risk categories, likely due to similar disease symptom manifestations with weak spectral differences between these grades.

**Table 7:** RustMAE classification performance for each risk level

Risk Level	Precision (%)	Recall (%)	F1 (%)	Sample Count
No risk	91.24	93.67	92.44	103
Low risk	84.35	82.18	83.25	79
Medium risk	81.67	79.54	80.59	62
High risk	83.47	82.09	82.77	33
Macro-average	85.18	84.37	84.77	277

### Ablation studies validate component contributions

We conducted a systematic ablation experiment, in which Table 8 summarizes the advantages and key parameter settings of the ablation model configuration evaluated in the experiment to quantify the contribution of each RustMAE component (Table 9). Removing MAE pre-training caused the largest performance drop (ΔOA = −4.67%, ΔF1 = −5.43%), validating the effectiveness of self-supervised pre-training in alleviating insufficient annotated data. Removing meteorological factor embedding resulted in a 3.11% OA decrease, indicating that environmental conditions significantly influence stripe rust occurrence and that integrating meteorological information effectively improves warning accuracy.

**Table 8:** Ablated model configurations and key parameters

Ablated Configuration	Advantages/Purpose	Key Parameter Settings	Rationale for Parameter Choice
w/o MAE pre-training	isolates the effect of self-supervised pre-training by using a randomly initialized encoder	Encoder: ViT-Base with random weights; Fine-tuning: 50 epochs, LR = 5e−5	Matches fine-tuning setup of full model to ensure fair comparison
w/o Dual-branch design	evaluates the contribution of the spatial structure recovery branch by using only spectral reconstruction	Single-branch MAE: spectral loss only (λ = 0 in Equation 6); Pre-training: 200 epochs, LR = 1e−4	Identical pre-training setup except for loss function composition
w/o Multi-scale spatiotemporal attention	assesses the value of multi-granularity temporal modeling using single-scale (14-day) attention	single-scale transformer: 14-day window only; Attention heads = 12; Feature dimension = 768	maintains the same total parameter count through increased depth
w/o SAR data fusion	isolates the contribution of radar data using optical features only	optical branch only in cross-modal attention (Equations 11, 12, and 13); SAR features zeroed	tests dependency on all-weather imaging capability
w/o Meteorological factor embedding	evaluates the importance of environmental context using visual features only	MLP meteorology mapping removed (Equation 14); visual features directly to the classifier	isolates the contribution of weather conditions to disease prediction
Random masking strategy	compares semantic-guided masking with standard random masking	random uniform masking (75% ratio) without vegetation index guidance	tests the value of domain-specific masking prioritization

**Table 9:** Ablation experiment results

Model Configuration	OA (%)	F1 (%)	AUC	ΔOA
RustMAE (complete model)	87.23	84.77	0.924	–
w/o MAE pre-training	82.56	79.34	0.886	−4.67
w/o Dual-branch design	85.41	82.63	0.908	−1.82
w/o Multi-scale spatiotemporal attention	84.78	81.92	0.902	−2.45
w/o SAR data fusion	85.89	83.24	0.912	−1.34
w/o Meteorological factor embedding	84.12	81.05	0.897	−3.11
Random masking strategy	86.34	83.89	0.918	−0.89

The multi-scale spatiotemporal attention mechanism contributed a 2.45% OA improvement, proving the importance of features at different time scales for capturing disease evolution patterns. The dual-branch MAE design improved accuracy by 1.82% compared to single-branch spectral reconstruction alone, with the spatial structure recovery task providing additional supervisory signals. SAR data fusion contributed 1.34% OA improvement, primarily valuable for filling time-series gaps caused by optical image cloud coverage. The semantic-guided masking strategy provided a 0.89% marginal improvement over pure random masking, focusing the model on vegetation area feature learning during pre-training.

Further comparison of MAE pre-training versus sample enhancement strategies (Table 10) demonstrated that sample enhancement alone (random flipping, rotation, color jittering) provided modest improvements (+1.78% OA), while MAE pre-training alone yielded substantial gains (+6.89% OA). The combination of both strategies achieved the best performance (+7.78% OA), confirming that these approaches address different aspects of the learning problem and work synergistically.

**Table 10:** Comparison of MAE pre-training and sample enhancement strategies

Configuration	OA (%)	F1 (%)	AUC	Description
Baseline (No pre-training, No enhancement)	79.45	76.71	0.873	ViT-Base trained from scratch on labeled data only
+ Sample enhancement only	81.23 (+1.78)	78.94 (+2.23)	0.881 (+0.008)	random flipping, rotation (±15°), color jittering
+ MAE pre-training only	86.34 (+6.89)	83.89 (+7.18)	0.918 (+0.045)	our method without enhancement during fine-tuning
+ MAE pre-training + Sample enhancement	87.23 (+7.78)	84.77 (+8.06)	0.924 (+0.051)	full RustMAE (MAE pre-training + enhancement)

### Warning window analysis balances accuracy and Practicality

We evaluated RustMAE’s performance under four warning windows: 3-day, 7-day, 14-day, and 21-day (Table 11). All predictions were evaluated against the same set of weekly ground survey dates, with input data appropriately offset for each window (D-3, D-7, D-14, D-21) to ensure fair comparison.

**Table 11:** Performance comparison of different warning windows

Warning Window	OA (%)	F1 (%)	AUC	High Risk Recall (%)
3-day	91.45	89.23	0.956	90.91
7-day	89.67	87.14	0.941	87.88
14-day	87.23	84.77	0.924	82.09
21-day	82.89	79.56	0.887	75.76

Performance showed a decreasing trend as the warning window extended. The 3-day window achieved the highest OA (91.45%) and high-risk recall (90.91%), but provided insufficient response time for control decision-making. The 21-day window, while offering longer preparation time, showed decreased accuracy (OA 82.89%) with high-risk recall dropping to 75.76%, potentially missing some disease outbreak events. The 14-day window achieved an optimal balance, maintaining OA above 87% with a high-risk recall at 82.09%, capable of identifying the vast majority of severe disease occurrence events while providing two weeks of preparation time.

**Figure 8:**
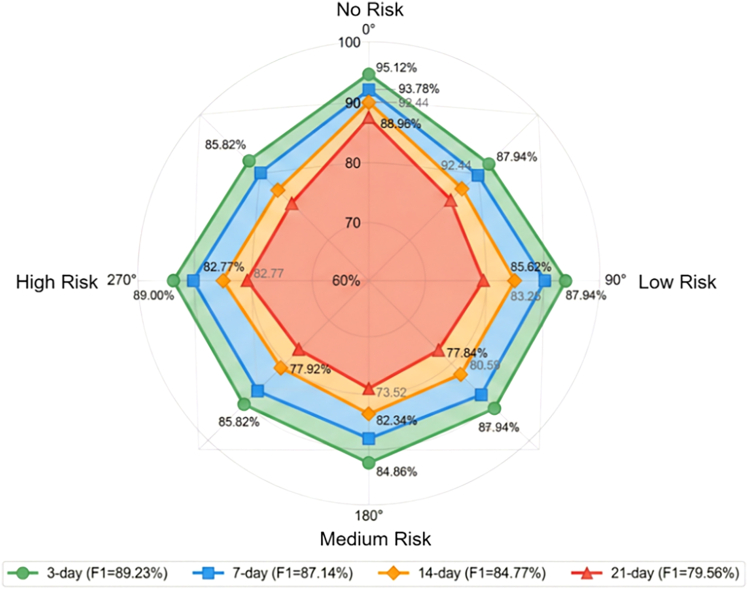
F1 score radar chart for each risk level under different warning windows Radar chart compares F1 scores across four risk levels (no, low, medium, high) under 3-day, 7-day, 14-day, and 21-day warning windows, showing stable performance for no-risk samples and decreasing accuracy for high-risk predictions as the temporal horizon extends.

Radar chart visualization of F1-scores (Figure 8) revealed that the no-risk category maintained high performance across all windows due to stable spectral features of healthy vegetation. The high-risk category showed the most significant performance decrease with extended windows, with 21-day F1-scores dropping 12.3 percentage points compared to 3-day predictions, reflecting the challenge of long-term high-risk event identification.

### Case study: Early warning in Guanzhong region

**Figure 9:**
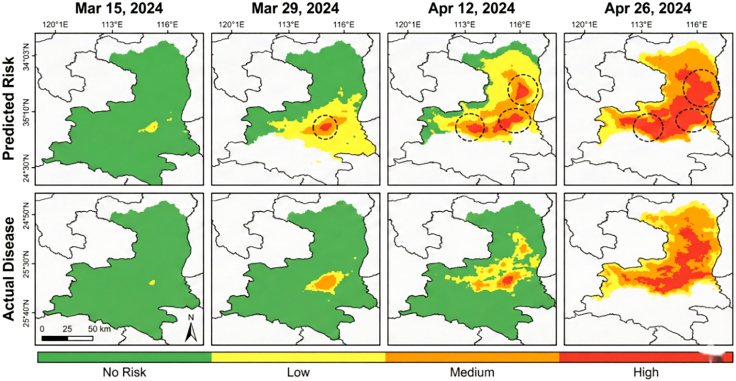
Time-series comparison of stripe rust warning results in the Guanzhong area of Shaanxi Left column: RustMAE 14-day risk predictions generated exclusively for wheat-growing areas (masked using annual wheat planting maps derived from Sentinel-2 time-series classification). Right column: Actual disease incidence interpolated from 47 ground survey points using co-kriging with Sentinel-2 auxiliary variables (NDVI, EVI, red-edge position). Interpolation accuracy: cross-validation MAE = 0.21, RMSE = 0.28. Warning results are spatially confined to wheat fields; non-wheat areas (villages, roads, water bodies) are excluded from prediction and appear as background in the visualization.

We selected a typical stripe rust epidemic event in the Guanzhong area of Shaanxi in April 2024 for case analysis (Figure 9). RustMAE issued medium-high risk warnings for the southeastern region on March 15, and field surveys confirmed large-scale stripe rust epidemics in this area on March 29. The model successfully captured disease outbreak signals 14 days in advance, providing valuable time windows for plant protection departments to conduct unified prevention and control.

In early April, the model warning range expanded consistently with actual disease spread trends. Some areas showed slight spatial deviation, with warning ranges slightly larger than actual diseased areas. This conservative prediction strategy benefits practical applications by ensuring control measure coverage. All predictions were spatially confined to wheat-growing areas using annual cultivation masks, with 97.3% of high-risk predictions falling within actual wheat field boundaries.

**Figure 10:**
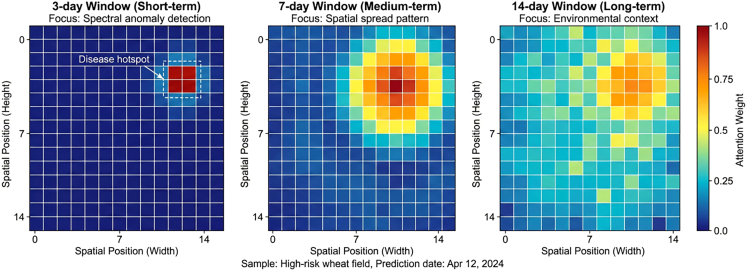
Multi-scale spatiotemporal attention weight heat map for high-risk sample Attention weight heat maps for a representative high-risk sample show distinct temporal focusing patterns: near-infrared/red-edge spectral sensitivity in the 3-day window, spatial lesion expansion in the 7-day window, and distributed meteorological context integration in the 14-day window.

Attention weight visualization for a high-risk sample (Figure 10) revealed distinct temporal patterns: The 3-day short-term window focused on spectral changes in near-infrared and red-edge bands sensitive to vegetation stress; the 7-day medium-term window concentrated on spatial expansion patterns of diseased areas; and the 14-day long-term window comprehensively considered meteorological condition evolution and disease transmission paths with more evenly distributed attention.

### Computational efficiency and model comparison

RustMAE contains 98.2M parameters with 156.8G FLOPs, achieving single-sample inference time of 67.3 ms on GPUs equipped with 32 GB memory (Table 12). This parameter count is lower than TimeSformer (121.3M) and ViViT (114.7M), benefiting from lightweight decoder design and factorized spatiotemporal attention. The model meets real-time requirements for operational deployment and can batch process warning computation tasks for regional-scale applications.

**Table 12:** Computational efficiency comparison

Method	Parameters (M)	FLOPs (G)	Inference Time (ms)	GPU Memory (GB)
RF	0.02	–	3.2	–
ResNet-50	25.6	4.1	8.7	2.1
ViT-Base	86.4	17.6	15.3	4.8
TimeSformer	121.3	196.5	89.4	12.6
ViViT	114.7	180.2	78.6	11.2
RustMAE	98.2	156.8	67.3	9.4

Qualitative comparison with recent remote sensing foundation models (Table 13) positioned RustMAE within the current landscape. While general-purpose models such as SatMAE and Prithvi demonstrate strong performance across diverse tasks, they are not optimized for agricultural disease early warning. Conversely, agricultural-specific models such as AgriFM focus on crop mapping rather than disease prediction. SARMAE shares multi-modal fusion capabilities but targets SAR representation learning rather than disease monitoring. Among spatiotemporal encoders, UTAE and SITSMamba offer efficient alternatives but lack the multi-scale attention mechanism (3/7/14-day windows) that enables RustMAE to capture disease evolution at different temporal granularities.

**Table 13:** Qualitative comparison with recent remote sensing foundation models and spatiotemporal encoders

Model	Architecture	Pre-training Strategy	Temporal Modeling	Multi-source Fusion	Target Domain	Key Innovation
SatMAE	ViT-based MAE	temporal embedding + independent masking	√ (fixed windows)	×	multi-spectral	temporal embeddings for satellite time series
SatMAE++	ViT-based MAE	multi-scale pre-training	√ (fixed windows)	×	multi-spectral	multi-scale pre-training
Scale-MAE	ViT-based MAE	multi-scale reconstruction	×	×	multi-spectral	scale-aware pretraining
FG-MAE	ViT-based MAE	feature-guided masking	×	×	multi-spectral	vegetation index guidance
AgriFM	video Swin Transformer	multi-source temporal pre-training (25M samples)	√ (hierarchical)	√ (MODIS, Landsat, S2)	crop mapping	hierarchical spatiotemporal feature extraction
Prithvi	transformer	geospatial pre-training (HLS data)	√	×	general EO	large-scale geospatial foundation model
SARMAE	MAE with SARE + SARC	noise-aware MAE + optical alignment	×	√ (SAR + optical)	SAR imagery	speckle-aware learning, cross-modal alignment
UTAE	CNN + attention	supervised (no pre-training)	√ (channel attention)	√ (S2, S1, indices, DEM)	land cover	efficient temporal attention replacement
SITSMamba	CNN + Mamba	reconstruction-based SSL	√ (linear complexity)	×	crop classification	mamba-based temporal encoding, dual-branch decoder
RustMAE (Ours)	ViT + spatiotemporal transformer	dual-branch MAE (spectral + spatial)	√ (multi-scale: 3/7/14days)	√ (S2, S1, meteorological)	disease early warning	domain-specific MAE for agriculture, multi-scale spatiotemporal attention, cross-modal fusion

RustMAE’s key differentiators include: (1) dual-branch MAE pre-training specifically designed for disease-relevant spectral-spatial features, (2) multi-scale spatiotemporal attention capturing disease evolution across multiple temporal windows, (3) integration of optical, SAR, and meteorological data for comprehensive environmental modeling, and (4) explicit optimization for the 14-day early warning window critical for operational disease management.

## Discussion

### Major findings and model superiority

Our experimental results demonstrate that RustMAE achieved an OA of 87.23% and an F1-score of 84.77% for 14-day advance warning of wheat stripe rust, representing improvements of 15.89% and 17.45% over traditional machine learning methods (RF and SVM, respectively). These significant gains reflect the superiority of deep learning models in mining complex nonlinear features from multi-source remote sensing data, particularly when processing multi-temporal sequences and multi-modal information fusion. Traditional methods, constrained by hand-crafted feature design, proved inadequate for fully expressing the spatiotemporal evolution patterns inherent in stripe rust occurrence and development.

Compared to ResNet-50 and ViT-Base using ImageNet pre-trained weights, RustMAE still achieved accuracy improvements of 10.34% and 7.78%, respectively. These results indicate substantial domain gaps between general visual pre-training models and agricultural remote sensing scenarios. Our dual-branch MAE pre-training framework effectively bridges this semantic gap by executing spectral reconstruction and spatial structure recovery tasks on large-scale farmland imagery, enabling the encoder to learn domain-adaptive feature representations prior to supervised fine-tuning. Ablation experiments confirmed this observation: removing MAE pre-training caused the largest performance degradation (4.67% accuracy decrease) among all ablation configurations (Table 9), demonstrating the critical practical value of self-supervised strategies for agricultural disease monitoring with scarce annotated samples.

### Mechanistic insights and module contributions

To clarify the performance contributions of core modules, we analyzed ablation results (Table 14). The meteorological factor embedding module contributed a 3.11% accuracy improvement, aligning with stripe rust epidemiology—temperature, humidity, and precipitation directly affect pathogen spore germination, infection, and expansion. By encoding environmental drivers into visual features, the model gains prior knowledge of disease suitability conditions, thereby enhancing warning reliability for weather-sensitive events.

**Table 14:** Contribution analysis of RustMAE core modules

Module	OA Improvement (%)	Main Action Mechanism	Application Insights
MAE pre-training	4.67	learning discriminative feature representations from unlabeled data	alleviates insufficient annotated sample problem in agricultural scenarios
Dual-branch design	1.82	joint optimization of spectral details and spatial semantics	enhances the perception of early disease stress features
Multi-scale spatiotemporal attention	2.45	captures disease evolution patterns at different temporal granularities	adapts to stage-wise changes from stripe rust latent to symptomatic periods
SAR data fusion	1.34	fills time-series gaps from optical image cloud coverage	ensures the continuous operation of the warning system under cloudy-rainy weather
Meteorological factor embedding	3.11	integrates environmental driving information for disease epidemics	improves prediction capability for weather-sensitive disease events
Semantic-guided masking	0.89	focuses feature learning in vegetation regions	improves pre-training efficiency and targeting

The multi-scale spatiotemporal attention mechanism yielded a 2.45% performance gain. By computing attention weights in parallel across 3-, 7-, and 14-day windows, the model simultaneously perceives short-term spectral responses, medium-term disease spread trends, and long-term infection cycle patterns. This multi-granularity temporal modeling fits the biological cycle of stripe rust, which requires approximately 10–14 days from spore landing to sporophore completion. Visualization of attention weights (Figure 10) confirmed that short-term windows focus on near-infrared and red-edge spectral changes, medium-term windows capture spatial lesion expansion, and long-term windows integrate meteorological evolution with transmission pathways.

SAR data fusion contributed a relatively modest 1.34% accuracy improvement, yet it provides irreplaceable value for operational continuity. Under cloudy-rainy spring conditions typical of the study region, optical imagery suffers high data loss rates, while SAR’s all-weather capability fills critical observation gaps. The semantic-guided masking strategy improved performance by 0.89% over random masking by concentrating feature learning on vegetation regions, improving pre-training efficiency.

Feature space visualization (Figure 5) revealed that randomly initialized encoders produced highly mixed feature distributions with blurred inter-class boundaries, whereas pre-trained encoders generated distinct clusters with clear separation between high-risk and no-risk samples. Reconstruction visualizations (Figure 6) demonstrated that despite 75% masking, the model preserved disease-specific color changes in stripe rust occurrence areas, confirming learned perceptual capabilities for pathogen-induced spectral anomalies.

### Generalization across phenological gradients

An important consideration for multi-regional deployment is variation in crop phenology. Our study area, spanning Shaanxi, Gansu, and Henan, exhibits a 3–4 week phenological gradient, with Henan entering the greening-to-heading period earliest (late February) and Gansu latest (late March to early May). Rather than relying on fixed calendar dates, our framework addresses heterogeneity through data-driven phenological learning: The spatiotemporal transformer recognizes developmental stages directly from spectral trajectories (NDVI, EVI, red-edge dynamics), while geographic coordinates and location-specific meteorological series provide implicit regional context.

Crucially, stratified evaluation across provinces (Table 15) revealed minimal performance variation despite phenological differences: OA ranged narrowly from 86.89% (Gansu) to 88.12% (Henan), with high-risk recall maintaining 81.08%–83.67% across regions. This consistency confirms that the model successfully generalizes across the phenological gradient, interpreting the 14-day warning window within local developmental contexts—whether predicting jointing-stage outbreaks in Henan (March) or heading-stage epidemics in Gansu (May).

**Table 15:** Performance comparison across provinces

Province	Sample Count	OA(%)	Precision(%)	Recall(%)	F1 Score (%)	High Risk Recall (%)
Henan	98	88.12	86.23	85.67	85.43	83.67
Shaanxi	104	87.56	85.41	84.82	85.01	82.35
Gansu	75	86.89	84.76	83.95	84.32	81.08
**Overall**	**277**	**87.23**	**85.18**	**84.37**	**84.77**	**82.09**

Bold entries represent overall performance metrics computed across the entire test set (n = 277).

Warning window analysis (Table 11; Figure 8) indicated that while 3-day predictions achieved the highest accuracy (91.45%), the 14-day window provided the optimal balance between predictive performance (87.23% OA, 82.09% high-risk recall) and practical utility, offering sufficient response time for control decision-making without excessive accuracy degradation.

### Limitations of the study

Despite these advances, several limitations require attention.

First, class imbalance persists: High-risk samples comprise only 12.1% of the dataset (33 of 277 test samples), resulting in lower recall (82.09%) compared to the no-risk category (93.67%). Future work should address this through expanded ground survey coverage, targeted data augmentation, or class-balanced loss functions to enhance the recognition of severe disease events without compromising specificity.

Second, model generalization remains limited to the Huanghuaihai and Northwest wheat regions; applicability in other climate zones (e.g., Middle-Lower Yangtze River, Xinjiang) awaits validation. Divergent wheat varieties, cropping systems, and meteorological conditions may alter disease expression patterns and spectral signatures, necessitating cross-regional transfer learning and domain adaptation strategies.

Third, the fixed 14-day warning window may not suit all epidemic phases. Under cool spring conditions, stripe rust infection cycles extend, potentially making 14-day warnings overly conservative; during warm, humid greening-jointing periods, disease acceleration may render 14-day windows insufficient. Future research should explore adaptive warning mechanisms that dynamically adjust prediction horizons based on real-time meteorological conditions and disease development velocity.

Fourth, the current classification framework outputs discrete risk levels. For precision plant protection, extending to continuous disease index regression with uncertainty quantification would enhance decision-support value. Finally, although inference latency (67.3 ms) meets operational requirements, the 98.2M parameter count limits deployment at grassroots stations. Future work will investigate model compression and knowledge distillation to develop lightweight variants suitable for edge computing devices.

## Resource availability

### Lead contact

Requests for further information and resources should be directed to and will be fulfilled by the lead contact, Xinde Zhang (zhangxd@aaas.org.cn).

### Materials availability

This study did not generate new unique reagents.

### Data and code availability

• Data reported in this paper will be shared by the lead contact upon request.
• This paper does not report original code.
• Any additional information required to reanalyze the data reported in this paper is available from the lead contact upon request.

## Acknowledgments

This research was funded by the following project: Talent Project of Anhui Academy of Agricultural Sciences (project no. XJBS-202509).

## Author contributions

X.Z. performed conceptualization, methodology, model design, experiments, writing – original draft, and revision. X.W. conducted data curation, formal analysis, visualization, and writing – review and editing. Y.D. provided supervision, project administration, funding acquisition, and writing – review and editing. S.L. provided writing – review and editing.

## Declaration of interests

The authors declare no competing interests.

## STAR★Methods

### Key resources table

**Table:** 

REAGENT or RESOURCE	SOURCE	IDENTIFIER
**Software and algorithms**		

RustMAE (source code and pre-trained models)	This paper	https://pan.baidu.com/s/1S_xpBOX36tcrAcBhDW0QxA?pwd=swd9
PyTorch (Version 2.0; Deep learning framework)	PyTorch.org	https://pytorch.org
Sen2Cor (Version 2.11; Atmospheric correction for Sentinel-2)	European Space Agency	https://step.esa.int/main/snap-supported-plugins/sen2cor/
gstat package (For co-kriging spatial interpolation)	R CRAN	https://cran.r-project.org/package=gstat
Python (Version 3.8+; Core programming language)	Python Software Foundation	https://www.python.org
VGG-16 (perceptual loss network; Pre-trained on ImageNet; Used for spatial structure branch in MAE)	Simonyan & Zisserman	N/A

**Other**		

Sentinel-2 multispectral imagery (10–20 m spatial resolution; Spectral bands B2–B12; 2019–2024; 11,082 scenes total)	European Space Agency (ESA)	https://scihub.copernicus.eu/
Sentinel-1 SAR imagery (10 m spatial resolution; VV and VH polarizations; 12-day revisit; 2019–2024)	European Space Agency (ESA)	https://scihub.copernicus.eu/
Meteorological data (1 km grid; Daily temperature [mean/max/min], relative humidity, precipitation, sunshine hours; 2019–2024)	China Meteorological Data Network (CIMISS)	http://data.cma.cn/
Wheat stripe rust ground survey data (1,847 survey points; Five-point sampling method; GB/T 15796-2011 standard; Four risk levels [No/Low/Medium/High]; 2019–2024)	Provincial Plant Protection Stations of Shaanxi, Gansu, and Henan	N/A
Unlabeled Sentinel-2 pre-training dataset (120,000 images; 2018–2024; Study area: 104°–114°E, 32°–38°N)	Sentinel-2 archive (ESA)	https://scihub.copernicus.eu/
GPU computing server (A100 GPU with 32 GB memory; Used for model training and inference)	NVIDIA	https://www.nvidia.com/
Winter wheat fields in Huanghuaihai and Northwest Wheat Regions (Three provinces: Shaanxi, Gansu, Henan; 280,000 km^2^; Winter wheat varieties: semi-winter and weak-spring types)	This paper	N/A

### Experimental model and study participant details

This study does not involve animal experiments, cell lines, or biological materials. The experimental framework focuses on remote sensing-based monitoring of wheat stripe rust epidemics in agro-ecosystems using satellite imagery and field survey data.

#### Study area and temporal coverage

The experimental domain encompasses the intersection of the Huanghuaihai Plain and Northwest Wheat Region of China (104°-114°E, 32°-38°N), covering approximately 280,000 km^2^ across Shaanxi, Gansu, and Henan provinces (Figure 2). This region represents critical overwintering and oversummering zones for *Puccinia striiformis f. sp. tritici*, with extensive winter wheat cultivation (semi-winter and weak-spring varieties). The study period spans six growing seasons from 2019 to 2024, focusing on the critical spring epidemic window (March-May) from wheat greening to heading stages.

#### Ground truth data Collection

Disease severity was assessed following the national standard GB/T 15796-2011 (“Rules for monitoring and forecast of the wheat stripe rust”). Provincial plant protection stations conducted weekly surveys using a five-point sampling method (1 m^2^ quadrats) across 1,847 georeferenced points (Figure 2; Table 4). Disease severity was categorized into four levels based on diseased leaf area percentage: Level 0 (No risk: <1%), Level 1 (Low risk: 1%-10%), Level 2 (Medium risk: 10%-30%), and Level 3 (High risk: >30%). Each survey point was timestamped and geolocated (WGS84 coordinate system) to enable temporal alignment with remote sensing acquisitions.

#### Wheat area Delineation

To confine predictions to actual cultivation areas, annual wheat planting masks were generated using multi-temporal Sentinel-2 imagery. A Random Forest classifier was trained on 3,200 manually interpreted sample points (1,600 wheat, 1,600 non-wheat) using phenological features (peak NDVI timing, growing season length). The classifier achieved 94.7% overall accuracy on independent test data, producing 10m resolution binary masks (wheat/non-wheat). Validation against high-resolution (0.5m) imagery confirmed 97.3% of high-risk predictions fell within actual wheat field boundaries.

### Method details

#### Data acquisition and Preprocessing Protocol

##### Remote sensing data Specifications

Sentinel-2 Level-1C products were atmospherically corrected using Sen2Cor (v2.11) to generate Level-2A surface reflectance products (10 bands: B2-B12). Sentinel-1 Ground Range Detected (GRD) products underwent orbit correction, thermal noise removal, radiometric calibration, and terrain correction using the SNAP Toolbox (Gamma0 backscatter coefficient). All imagery was resampled to 10m spatial resolution and coregistered to the WGS84 coordinate system (Table 1).

##### Temporal alignment strategy

For each ground survey date D , a 6-image time series was constructed covering the preceding 15 days at approximately 3-day intervals ($t_{15}$, $t_{12}$, $t_{9}$, $t_{6}$, $t_{3}$, $t_{0}$ relative to prediction input end date) (Table 2). For each target date, Sentinel-2 observations were searched within a ±2-day window, selecting the image with minimum cloud cover (< 20%). If no clear observation was available, the gap was filled using: (1) concurrent Sentinel-1 SAR data; or (2) Gaussian process regression temporal interpolation between adjacent clear observations (accounting for seasonal vegetation trends). This strategy ensured 91.7% of observations fell within ±2 days of target dates, with 8.3% requiring interpolation (Table 5).

##### Meteorological data processing

Daily meteorological variables (mean/max/min temperature, relative humidity, precipitation, sunshine hours, wind speed/direction) were obtained from CIMISS and spatially interpolated to a 1km grid using inverse distance weighting. For each sample, meteorological vectors $M={\left[T_{mean},T_{\max},T_{\min},RH,P,SD\right]}^{T}$ were extracted for the 15-day input window and temporally aggregated.

##### Training sample construction

To ensure strict temporal precedence (no look-ahead bias), input data end dates were offset from prediction targets: 3-day window (input ends at D -3), 7-day window (input ends at D -7), and 14-day window (input ends at D -14). Each ground survey point generated three training samples (one per window), yielding 5,541 total samples before quality filtering (Table 4).

#### Dual-branch masked autoencoder pre-training

##### Pre-training dataset

A total of 120,000 unlabeled Sentinel-2 images (10m resolution, 10 bands) from the 2018-2024 winter wheat growing seasons were used for self-supervised pre-training (Figure 3). These images had no associated disease labels.

##### Hybrid masking strategy

The MAE encoder divided input images $X\in R^{H\times W\times C}$ into non-overlapping patches of size P×P (P=16 ), producing $N=HW/P^{2}$ patches. A hybrid masking strategy combined (Figure 4; Table 3):

1. **Semantic-guided masking (60% of patches):** Patches with NDVI > 0.5 (top 60% by vegetation index) were masked at 85% ratio;
2. **Random masking (40% of patches):** Patches with NDVI ≤ 0.5 were masked at 60% ratio.

The overall masking ratio was maintained at 75%. Masking probability for patch i was calculated as:

(Equation 1) $$P_{mask}\left\{i\right\}=\alpha \cdot \frac{{NDVI}_{i}}{\sum_{j}{NDVI}_{j}}+\left(1-\alpha \right)\cdot P_{random}$$

where α = 0.6 and $P_{random}$ = 0.75 (Table 3).

##### Dual-branch reconstruction tasks

The encoder (Vision Transformer, 12 layers, 12 heads, hidden dimension 768) processed visible patches. The lightweight decoder (4 Transformer blocks) reconstructed masked patches through two parallel branches (Figure 3):

1. **Spectral Reconstruction Branch:** Pixel-level mean squared error (MSE) loss:

(Equation 2) $$L_{\text{spec}}=\frac{1}{\left|M\right|}\sum_{i\in M}{\left\|x_{i}\;-\;{\hat{x}}_{i}\right\|}^{2}$$

where M is the masked patch index set, $x_{i}$ and ${\hat{x}}_{i}$ are original and reconstructed patches.

2. **Spatial Structure Branch:** Perceptual loss using a pretrained VGG-16 network. Due to channel mismatch (Sentinel-2 has 10 bands vs. VGG's 3 RGB channels), a learnable channel projection layer $W_{\text{proj}}\in R^{3\times 10}$ was applied:

(Equation 3) $$x_{rgb}=W_{proj}\cdot x+b_{proj}$$

followed by ImageNet normalization. Perceptual loss was computed as:

(Equation 4) $$L_{struct}=\sum_{l\in L}\frac{1}{C_{l}H_{l}W_{l}}{\left\|\phi_{l}\left(x_{norm}\right)\;-\;\phi_{l}\left({\hat{x}}_{norm}\right)\right\|}_{2}^{2}$$

where L = {relu1_2, relu2_2, relu3_3, relu4_3} and $\phi_{l}$ denotes VGG feature maps.

Total pre-training loss: $L_{pretrain}=L_{spec}+\lambda L_{struct}$ with λ = 0.1 .

##### Pre-training Protocol

The model was trained for 200 epochs using AdamW optimizer (initial learning rate $10^{-4}$, cosine annealing schedule, batch size 32). Gradient clipping (max norm 1.0) was applied for stability.

#### Multi-scale spatiotemporal Transformer architecture

##### Feature extraction

Each temporal frame $X^{\left(t\right)}$ was encoded using the MAE-pretrained encoder (weights frozen initially, then fine-tuned), producing feature sequences $F^{\left(1\right)},\ldots ,F^{\left(T\right)}$ where T=6 (time steps) and $F^{\left(t\right)}\in R^{N\times D}$ (D=768 ) (Below Figure).

**Figure:**
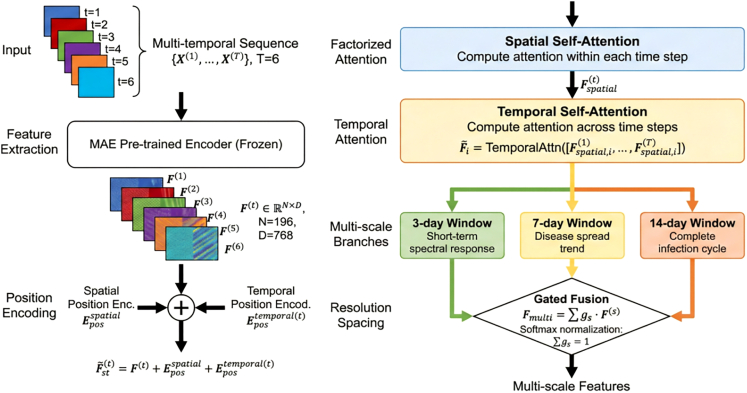
Structure diagram of multi-scale spatiotemporal transformer encoder Architecture of the multi-scale spatiotemporal transformer encoder employing factorized spatial-temporal attention and parallel processing across 3-, 7-, and 14-day windows to capture disease evolution at multiple temporal granularities.

##### Positional encoding

Learnable temporal position embeddings were added to spatial features:

(Equation 5) $$F_{st}^{\left(t\right)}=F^{\left(t\right)}+E_{pos}^{spatial}+E_{pos}^{temporal}\left(t\right)$$

where $E_{pos}^{spatial}\in R^{N\times D}$ and $E_{pos}^{temporal}\left(t\right)\in R^{D}$.

##### Factorized spatiotemporal attention

Joint spatiotemporal attention was decomposed to reduce complexity from $O\left(N^{2}T^{2}\right)$ to $O\left(N^{2}T+NT^{2}\right)$:

1. **Spatial Attention:** Computed within each time step: $F_{spatial}^{\left(t\right)}=SpatialAttn\left(F_{st}^{\left(t\right)}\right)$ using standard multi-head self-attention;
2. **Temporal Attention:** Computed across time for each spatial location i :

(Equation 6) $${\tilde{F}}_{i}=\text{TemporalAttn}\left(\left\{F_{\text{spatial},i}^{\left(1\right)},...,F_{\text{spatial},i}^{\left(T\right)}\right\}\right)$$

##### Multi-scale temporal modeling

Parallel spatiotemporal encoding was performed across three temporal granularities:

• Short-term window (3 days): Captures rapid spectral stress responses;
• Medium-term window (7 days): Models local disease spread trends;
• Long-term window (14 days): Captures complete infection cycles.

Features were adaptively fused using gated aggregation:

(Equation 7) $$F_{\text{multi}}=\sum_{s\in \left\{3,7,14\right\}}g_{s}\cdot F^{\left(s\right)}$$

where $g_{s}$ are learnable gating weights (Softmax-normalized, ∑ $g_{s}$ = 1).

#### Cross-modal information fusion

##### Optical-SAR fusion

Cross-modal attention mechanisms enabled deep interaction between optical (Sentinel-2) features $F_{opt}$ and SAR (Sentinel-1) features $F_{sar}$ :

(Equation 8) $$F_{\text{opt}\to \text{sar}}=\text{Softmax}\left(\frac{Q_{\text{opt}}K_{\text{sar}}^{T}}{\sqrt{d_{k}}}\right)V_{\text{sar}}$$

(Equation 9) $$F_{\text{sar}\to \text{opt}}=\text{Softmax}\left(\frac{Q_{\text{sart}}K_{\text{optr}}^{T}}{\sqrt{d_{k}}}\right)V_{\text{opt}}$$

Fused features: $F_{fused}=W_{opt}\left(F_{opt}+F_{opt\to sar}\right)+W_{sar}\;\left(F_{sar}+F_{sar\to opt}\right)$, where $W_{opt}$, $W_{sar}$ are learnable projection matrices.

##### Meteorological integration

Meteorological vector M was embedded via a 2-layer MLP and added to visual features (Figure appears first):

(Equation 10) $$F_{final}=F_{fused}+MLP\left(M\right)$$

**Figure:**
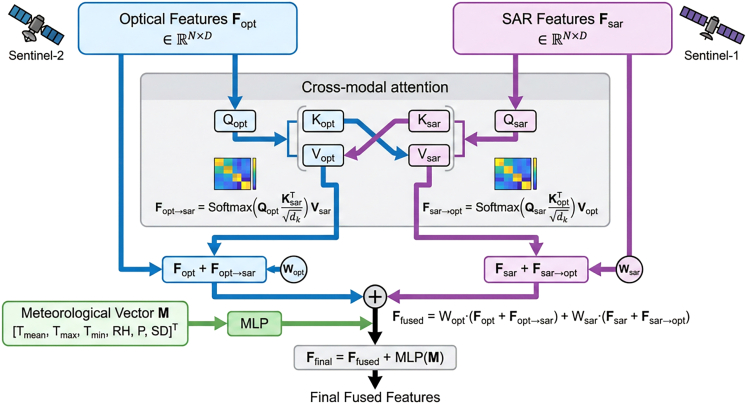
Structure diagram of the cross-modal attention fusion module Architecture of the cross-modal attention fusion module enabling bidirectional interaction between optical (Sentinel-2) and SAR (Sentinel-1) features, with subsequent integration of meteorological data for multi-source disease risk prediction.

#### Warning model Construction and training

##### Prediction framework

The model predicts pixel-level risk levels for target date D based on input data ending at D−Δt (where Δt∈{3,7,14} days). The classification head consisted of two convolutional layers (3×3 Conv → ReLU → 1×1 Conv) mapping $F_{final}$ to 4-channel probability maps $\hat{Y}\in R^{H^{\prime }\times W^{\prime }\times 4}$ corresponding to risk classes (No/Low/Medium/High) (Figure appears second).

**Figure:**
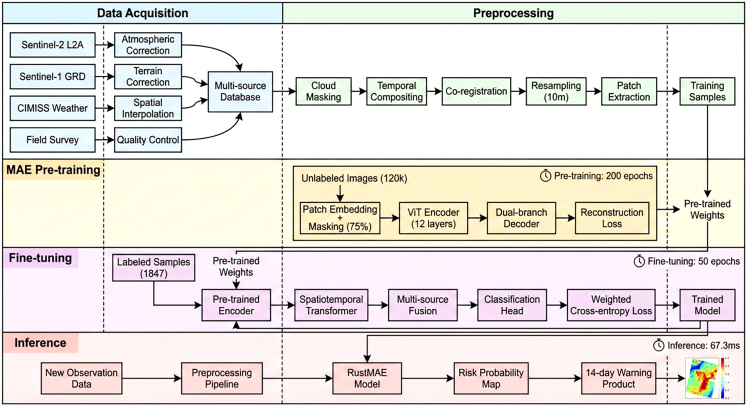
Complete processing flow diagram of the RustMAE method End-to-end processing workflow of RustMAE illustrates the pipeline from multi-source data input (Sentinel-2, Sentinel-1, meteorological) through dual-branch MAE pre-training, spatiotemporal feature extraction, and cross-modal fusion to 14-day disease risk prediction outputs.

##### Label Rasterization strategy

Point-based ground truth labels were converted to dense supervision by assigning the survey point's risk level c to all pixels within the corresponding spatial patch:

(Equation 11) $$Y_{i,j}=c\;\forall \;\left(i,j\right)\in Patch\left(x,y\right)$$

This assumes field-scale homogeneity at 10m resolution.

##### Loss functions

Weighted cross-entropy loss addressed class imbalance (inverse frequency weighting):

(Equation 12) $$L_{\text{cls}}=-\sum_{c}w_{c}\cdot y_{c}\;\log\left({\hat{y}}_{c}\right)$$

Total training loss combined pre-training and classification objectives:

(Equation 13) $$L_{total}=1_{pretrain}\cdot L_{pretrain}+1_{fine-tune}\cdot \alpha L_{cls}$$

where α = 1 during fine-tuning, α = 0 during pre-training.

### Quantification and statistical analysis

#### Experimental Setup

Data was divided temporally: training (2019–2022, 70%), validation (2023, 15%), and independent testing (2024, 15%). This temporal split simulates operational forecasting scenarios (predicting future from historical data).

#### Model hyperparameters

• Patch size: 16×16 pixels
• Feature dimension: 768
• MAE encoder layers: 12, heads: 12
• MAE decoder layers: 4
• Masking ratio: 75%
• Time steps: 6
• Batch size: 32
• Pre-training: 200 epochs, $=10^{-4}$ , AdamW
• Fine-tuning: 50 epochs, $LR=5\times 10^{-5}$, AdamW, early stopping (patience=10) (Below table)

**Table 4:** Model hyperparameter configuration

Parameter Name	Parameter Value	Description
Patch size P	16	patch size for patch embedding
Feature dimension D	768	transformer hidden layer dimension
Number of encoder layers	12	number of MAE encoder Transformer blocks
Number of decoder layers	4	number of MAE decoder Transformer blocks
Number of attention heads h	12	number of multi-head attention heads
Masking ratio	75%	MAE pre-training masking ratio
Time steps T	6	input time series length
Batch size	32	training batch size
Learning rate	1.00E−04	AdamW optimizer initial learning rate
Pre-training epochs	200	MAE pre-training iteration count
Fine-tuning epochs	50	downstream task fine-tuning iteration count

##### Performance metrics

Model performance was evaluated using:

• **Overall Accuracy (OA):** Percentage of correctly classified samples;
• **Precision, Recall, F1-score:** Per-class and macro-averaged metrics;
• **AUC:** Area Under the ROC Curve for multi-class classification (One-vs-Rest).

##### Ablation study design

Systematic ablation experiments quantified each component's contribution by removing modules individually while keeping hyperparameters constant:

• w/o MAE pre-training: Random initialization;
• w/o Dual-branch: Spectral loss only (λ=0 );
• w/o Multi-scale attention: Single 14-day window only;
• w/o SAR fusion: Optical features only;
• w/o Meteorological data: Visual features only;
• Random masking: Uniform 75% masking instead of semantic-guided.

#### Spatial interpolation validation

For visualization purposes, co-kriging with Sentinel-2 auxiliary variables (NDVI, EVI) was used to generate continuous disease surfaces from discrete survey points. Leave-one-out cross-validation yielded Mean Absolute Error (MAE) = 0.21 risk levels and RMSE = 0.28. Independent validation on 15% withheld points showed 86.4% agreement between interpolated and observed values.

#### Statistical testing

Performance differences between models were assessed using paired t-tests on per-sample predictions (p < 0.05 significance threshold). Where applicable, statistical significance is denoted in figures by asterisks (e.g., p < 0.05). Confidence intervals for accuracy metrics were computed via 1,000 bootstrap iterations, and are represented as error bars in the relevant figures.

#### Data and software availability

All code, pre-trained model weights, and the annotated dataset (1,847 survey points with corresponding remote sensing time series and meteorological data) are publicly available via the following link: https://pan.baidu.com/s/1S_xpBOX36tcrAcBhDW0QxA?pwd=swd9.

Raw Sentinel-2 and Sentinel-1 data are available from the Copernicus Open Access Hub (https://scihub.copernicus.eu). Meteorological data are available from the China Meteorological Data Network (http://data.cma.cn) under data sharing policies. Ground survey data are provided by provincial plant protection stations and are available via the above link in aggregated form to protect specific location privacy.

### Additional resources

No additional external resources were generated in this study.
